# Cooperate! A paradigm shift for health equity

**DOI:** 10.1186/s12939-016-0508-4

**Published:** 2017-02-21

**Authors:** Wei-Ching Chang, Joy H. Fraser

**Affiliations:** 1 Department of Medicine, University of Alberta, Edmonton, Alberta Canada; 2 Health Administration Program, Athabasca University, Edmonton, Alberta Canada

**Keywords:** Competition, Capitalism, Ethical fading, Cooperation, the cooperative movement, Paradigm shift, Health equity

## Abstract

The role of competition and cooperation in relation to the goal of health equity is examined in this paper. The authors explain why the win-lose mentality associated with avoidable competition is ethically questionable and less effective than cooperation in achieving positive outcomes, particularly as it relates to health and health equity. Competition, which differentiates winners from losers, often with the winner-takes-all reward system, inevitably leads to a few winners and many losers, resulting in social inequality, which, in turn, engenders and perpetuates health inequity.

Competitive market-driven approaches to healthcare—brought about by capitalism, neo-liberalization, and globalization, based primarily on a competitive framework—are shown to have contributed to growing inequities with respect to the social determinants of health, and have undermined equal opportunity to access health care and achieve health equity. It is possible to redistribute income and wealth to reduce social inequality, but globalization poses increasing challenges to policy makers. John Stuart Mill provided a passionate, philosophical defense of cooperatives, followed by Karl Polanyi who offered an insightful critique of both state socialism and especially the self-regulating market, thereby opening up the cooperative way of shaping the future. We cite Hannah Arendt’s “the banality of evil” to characterize the tragic concept of “ethical fading” witnessed in business and everyday life all over the world, often committed (without thinking and reflecting) by ordinary people under competitive pressures.

To promote equity in health for all, we recommend the adoption of a radically new cooperation paradigm, applied whenever possible, to everything in our daily lives.

## Background

*History is likely to judge the progress in the 21st century by one major yardstick: is there a growing equality of opportunity between people and among nations?* (*Human Development Report* 1995: p. iii)

The notion of health as a human right is central to the creation of equitable health systems [1, 2]. The right to health equity has been reflected globally in national constitutions, treaties and domestic laws, policies, and programs, and is included as a priority item in United Nations’ post-2015 sustainable development agenda [3–5]. In 2000, the International Society for Equity in Health (ISEqH) was formed, and held its inaugural conference, to promote health equity, where Chang [6] presented a paper explaining the meaning and goals of equity in health, promoting equal opportunities to actualize optimal health for all. Jackson and Huston ([7], p. 19) recently reiterated that “the goal of working on health equity and determinants of health is to improve the health of the population and to ensure that the conditions that support health are distributed fairly.”

Yet, despite good intentions, these goals remain elusive, as seen by the persistence of large disparities in health both within and between countries, and the growing disparities between poor and rich countries [8, 9]. Even in affluent countries, extreme income and social inequalities have led to social failure, as manifested in the prevalence of drug abuse, obesity, cardiovascular disease, anxiety, depression, teenage pregnancies, violence and imprisonment [10]. We contend that, although numerous researchers have traced the causes of health inequity to social inequality, policy makers have been reluctant to take the next step and identify ‘the competition paradigm’ as the true culprit of social inequality. To make real and sustainable progress toward health equity, we submit that we must go further upstream to re-evaluate the role of competition in exacerbating social inequality and, therefore, health inequity. In addition, we must propose an alternative vision and a roadmap to lead us toward the goal of health equity.

It should be noted that, throughout history, humans have been compelled to compete, fight, and win in order to survive or gain power. While some forms of competition are unconscious and unavoidable, the focus of this paper is on those human conflicts that are conscious, unnecessary and avoidable. While the winners of competition reap the rewards, the losers are disgraced or worse. Most conflicts have been resolved through coercion, violence, and war to overpower the competitors, human and non-human, whether in the fields of science, technology, business and economics, or sports and entertainment. The domination of competition in our lives, and particularly in the United States, is depicted by Pauline Rosenau as follows ([11‬‬‬‬‬‬‬‬‬‬‬‬‬‬‬‬‬‬‬‬‬‬‬], p.5)

The competition paradigm takes on an almost moral stance in America today. If some competition is good, more competition is better. Winning is not just valued, it is a virtue. Competition becomes a builder of character, a test of personal worth, and a powerful stimulus to individual achievement that ultimately produces the maximum economic value for society.

Competition is indeed embedded in our current way of life; however, we will show, as Deutsch [12] also concluded, that competition tends to generate negative power relationships, and is therefore antithesis to the vision of health equity.

The purpose of this paper is two-fold: 1) to explain why our hegemonic, win-lose mentality and unnecessarily competitive orientation leads us away from health equity, and 2) to propose an alternative cooperative orientation at the personal, organizational, and governmental policy levels, as a precondition for moving closer to the ideal of equity in health.

### Competition is unhealthy and immoral

*If competition remains the means by which individuals will or will not survive, then this social contract creates incentives for individuals to gain advantage over fellow members. This contract benefits the individual desirous of more than an equal share of material goods…(Stephen Faison, Philosophy Now, 2016; 116, Oct/Nov: p.15)*

Our central thesis is that most forms of competition lead to behaviours that are unhealthy and immoral. Why? Competition is specifically designed to separate winners from losers, with only few winners but plenty of losers. Rewarding only the winners and not the losers leads to a focus on winning rather than on doing well, or ‘doing good’. Winning becomes an obsession, the only thing that matters. Competition drives a wedge among us, as it engenders jealousy and resentment, secrecy and distrust, superiority and inferiority complexes, the haves and the have-nots, and the rich and the poor; it increases inequalities in all spheres of our lives. Losing, therefore, leads to antipathy, depression, violence, war, and increased aggression on all fronts [13–17]. Since competition means that one person can succeed only if others fail, it follows that even for winners, it is clearly immoral to feel good for beating another, thus turning the winners into sadists, knowingly or not.

#### Constructive competition

Rosenau observed that “when competition is constructive, it involves competing at efficiency in controlled circumstances.” Therefore, “enthusiastic about competition in principle,” she wrote that “most people enjoy competition at some level, be it card games or basketball. These forms of competition do not do much harm if they are not taken too seriously” ([11], p.10.) In reality, many people do approach these forms of competition less constructively and collaboratively, and in the end, they often become antagonistic as we witness in sports among players, and among fans during and after sporting events.

In 2009, Hague [16] observed “20th century competition hinged on the idea that unbridled greed, naked self-interest, and coercion were the essential drivers of growth. But last year’s market collapse demonstrated the fundamental incompatibility of those ideas with an interdependent world.” He noted further that the most haunting example of unethical practices “is pharma itself: by lobbying hard for subsidies and patent enforcement, what strategic outcome did pharma incumbents realize? A deluge of global low-cost hypercompetition, that has left incumbents shocked, stunned, and stumbling.” Thus, Hague argued for the next hundred years the promotion of *constructive competition* based on ethical practices, which he characterizes poetically as follows:

Fair is fair, and foul is foul. Avarice and usury are yesterday’s fallen idols, and peace, equity, and meaning are our new gods. How much can we change the world radically for the better?

Fülöp found that when individuals compete “[in] a constructive competitive process, the means of competition can be cooperation, helping, and sharing. These are characteristic of competition between friends.” ([17], p.143). Nevertheless, as Fülöp later found, even in friendly competitions, winning and losing both tend to evoke positive (e.g., happiness, pride, increased motivation for the future, learning about the self) and negative (e.g., guilt, embarrassment, sadness, anger, shame) emotions. She concluded that competition can be either a friendly process or “a desperate fight full of aggression among the competitors who consider each other enemy”, producing “anxiety provoking, stressful, and exhausting negative experience that leads to interpersonal conflicts and has destructive consequences individually, to the group and ultimately to the society.” ([18], p.345). For competition to be constructive, therefore, the competitors have to act cooperatively, helping each other to reach a common, person-specific, or higher goal so all can win, and avoid the slippery slope of degenerating into the so-called “destructive competition”. Thus, ‘constructive competition’ has to be a form of genuine cooperation without creating a 'sore' loser.

#### Ethical fading

There is a plethora of literature showing how, even when people start out following fair and ethical rules of conduct, competitive pressures eventually prompt the trampling of ethical considerations. As a result, ethical decision-making is often compromised, resulting in cheating, bribery, corruption, excessive executive pay, corporate earning manipulation, commercialization of university research, child labour, prostitution, and other immoral acts [19–26]. Tenbrunsel and Messick called this phenomenon “ethical fading”—taking ethics out of consideration or even enhancing unethical behaviour [26]. The title of Schurr and Ritov’s paper underscores the issue: “Winning a competition predicts dishonest behaviour.” [23] These authors noted that while competition plays an important role “in advancing economic growth, technological progress, wealth creation, social mobility, and greater equality,” their research showed that “winning a competition engenders subsequent unrelated unethical behavior” ([23], p.1754). They go on to surmise that this tendency toward unethical behavior on the part of winners is likely in the long run, to exacerbate societal disparities in society, rather than alleviating them.

The ethical fading exhibited by health professionals or industry partners competing for market share, has serious, widespread, harmful effects. Fraser [27] and Lexchin [28] have described numerous cases where the pharmaceutical industry has used unethical measures, such as suppressing study results disadvantageous to marketing goals, or choosing trial designs and the selection of trial participants that favor a targeted drug, to bias the outcomes of clinical trials of medications. Industry-paid physicians and pharmacists then write research articles with the “editorial assistance” of industry-paid writers, careful to report only selected, favourable study outcomes [28]. Though it is a clear conflict of interest, pharmaceutical manufacturers sponsor the publication of multiple reviews, commentaries, letters, and case reports to create the impression that a targeted drug is more effective or safer than what is supported by science. This distorted information, once ensconced in the medical literature, is propagated by industry and by well-intentioned authors who unwittingly cite these studies. The impact of ethical misconduct on the part of the sponsors, researchers, and authors of medical research and publications is damaging, not only to evidence-based practice, but ultimately also to patients, and society at large.

#### Social inequality

Competition is a sure way to exacerbate social inequality at all levels, whether it involves individuals, groups, organizations, business entities, regions, or nations. As Rosenau stated ([11], p. 6)﻿,

Under conditions of intense competition, results are predictable. This is because at the outset, competitors seldom start at the point of equality. Some have more resources, attributes, and wealth than others…. The most destructive forms of competition increase these differences and sustain a spiral of winning and losing, thus generating even greater levels of inequality. Eventually, and in the absence of any outside interventions…it leads to big winners and continual losers…Repeated losers, be they individuals, organizations, or societies, make for lower overall societal productivity. In the end, everyone is worse off because when productivity suffers, the quality of life is compromised for all.

Unsurprisingly, Oxfam [29] reported in January 2016 that: “The richest 1% now have more wealth than the rest of the world combined” and “62 people own as much wealth as the poorest half of the world’s population.” These findings have been corroborated by the French economist Thomas Piketty and his associates [30, 31], after analysing massive income tax data covering periods from 15 years (China) to 132 years (Norway), and 22 countries in Europe, North America, Australia and New Zealand, Latin America, and Asia. They concluded that income and wealth inequality, is a feature of capitalism, and will tend to increase without limit in the absence of government interventions.

#### Health inequity

There is a wealth of research demonstrating the relationship between social inequality and health inequity [32–36]. The findings of the 2015 Canadian Institute for Health Information report entitled *Trends in Income-Related Health Inequalities in Canada* are typical ([32], p.7﻿),

Our analysis identified that there has been minimal progress in reducing the health gap between lower- and higher-income Canadians over the past decade. For the majority of indicators, this gap has persisted or widened over time.

In fact, this report identified increased inequality beginning in the mid-1990s, due to a larger income increase in the highest income level than in the lowest income level ([32], p.33).

In a comprehensive study of the impact of social inequality on social and individual health, Wilkinson and Pikett [10] researched the 23 most affluent counties of the world based on data from the United Nations, the World Bank, the World Health Organization and the US Census. They found that inequality has pernicious effects on societies, eroding trust, increasing anxiety and illness, and encouraging excessive consumption. They found it ironic and paradoxical that material success in countries such as the US and the UK comes with significant social failure: diminished community life and social relations, lower life expectancy due to the prevalence of drug abuse and other physical (e.g., obesity and cardiovascular disease) and mental health (e.g., anxiety and depression) problems, teenage pregnancies, violence and imprisonment, lower educational performance, and limited social mobility. Their research showed that this paradox could only be reasonably explained by social inequality associated with these competitive societies, where what matters is where we stand in relation to others: our social status and relative income. More equal societies such as Japan, Singapore, Sweden and Norway seem to fare much better psychosocially: people tend to be more community-oriented, healthier and more environmentally responsible. Wilkinson and Pikett’s inescapable conclusion: income inequality is linked to social dysfunction. Their simple message: we do better when we are equal.

A sobering economic implication of Wilkinson and Pickett’s research is that inequality is costly: it increases the need for big government—for more health and social services, and for more police and prisons. These public programs are very expensive to fund and operate, and yet only partially effective, with little prospect for improvement in cost-effectiveness. Wilkinson and Pikett therefore surmised: “In fact, one of the best and most humane ways of achieving small government is by reducing inequality.” ([37], p. 295).

Some may claim that the afore-mentioned extreme concentration of wealth need not have happened were we to enact just tax policies, redistributing incomes from the wealthy to the poor. In the following sections, therefore, we will address the questions: 1) Can we make competition more constructive within a competitive paradigm? 2) Should the competition paradigm be defended? 3) How can we move more toward a co-operative paradigm?

### Making competition more constructive

An obvious way to make competition more constructive is to modify the winner-takes-all incentive system so that some of the rewards are shared with the losers. For instance, governments could institute a progressive income and capital tax in order to redistribute money from the rich to the poor, the strategy studied in detail by Samuel Bowles and his associates. They justified egalitarian measures, disputing the conventional efficiency-equity trade-off argument that the pursuit of equity objectives would impair productivity and hence lower the living standards, stating: “More egalitarian distributions are likely to be more efficient. The reason is that it is the poor, not the wealthy, who are precluded from engaging in efficient contacts.” ([38], p.70). According to these economists, one of the key considerations for such a measure is that it should enhance productivity. Since income-based strategies are rarely better and are often worse than productivity-neutral strategies, asset-based measures are preferred because they can, in principle, enhance productivity. Moreover, redistributing assets not only addresses a major cause of unequal income, but also leaves the market to do the job of identifying ‘losers’ and getting them out of the game.

Globalization, however, makes it extremely challenging, if not politically impossible, for a national government to design and implement a redistribution strategy that would not depress the expected after-tax rate of return to capital, or to alter the relative prices of tradable goods and services. Bowles observed that one of the reasons is

…that the more internationally mobile factors of production—capital and professional labor—tend to be owned by the rich, and a nation-specific tax on a mobile factor induces national-output-reducing relocations of these factors ([38],p. 74).

In view of the possible flight of capital along with highly-skilled professionals, egalitarian redistribution in an open economy is feasible only by: (1) increasing productivity, as was done in Sweden and Singapore; (2) reducing costs, as in the case of co-operatives and mutuals; or (3) redistributing labour income without eroding work incentives, as might be accomplished by guaranteed annual income. The ideal policy, however, would be a progressive global tax on capital, as suggested by Piketty: “Such a tax is the only way of democratically controlling this potentially explosive process while preserving entrepreneurial dynamism and international economic openness.” ([36], p.444). Recognizing that it is a utopian ideal, he proposed a regional or continental tax for countries willing to participate voluntarily. Since a high degree of international co-operation would be required, a paradigm shift toward greater international co-operations would be necessary to carry out such a policy.

### Is competition (In Healthcare) defensible?

In view of the challenges we may face in making competition more constructive, is it possible to defend competition at all, not only in terms of health equity and morality, but in terms of its superiority over cooperation in quality, efficiency and cost? Writing in the *British Medical Journal* in 2007, Charlton decried “the doctrinaire anti-capitalism characteristic of public health administrators, including the World Health Organization.” He praised capitalism (and indirectly, competition) for producing the “largest scale reduction of poverty in the history of the planet” in China, India, etc. in recent decades. Charlton further asserted that “China alone is lifting a million people a month out of poverty.” ([39], p.628). There is no denying that capitalism, with its relentless, competitive orientation, has stimulated economic growth in these countries, but it has also exacerbated social inequality. Therefore, let us examine more closely the impact of competition in the health care sector.

Although empirical evidence is scarce in this regard, studies on competition by Cookson and colleagues [40–42], showed that socioeconomic equity in the use of healthcare services had not been compromised in the context of English National Health Service with universal health care. As Cookson et al. explained: “This may be because the ‘dose’ of competition was small and most hospital services continued to be provided by public hospitals which did not face strong incentives to select against socioeconomically-disadvantaged patients.” ([41], p.55).

On the other hand, Bevan and Skellern [43] reported that there is a lack of clear evidence of any benefit from inter-hospital competition in the NHS. These researchers undertook a comprehensive review of the research and the debates on the NHS, focussing on the effects of hospital competition on quality of care within the English NHS, rather than solely on the costs of competition (such as transaction costs). They concluded that much of the published research claiming the positive effects of competition is flawed, and in fact it leaves more questions than answers. For one thing, the NHS studies had not addressed the issue of “how might quality of care be improved in rural areas where competition is unalterably weak, or for types of care for which it is more difficult to design effective competition?” ([43], p. 943). Furthermore, questions related to the cost effectiveness of competition and how it compares with other policies for increasing hospital quality remain unanswered. Bevan and Skellern therefore cautioned against plans to further extend competition.

Interestingly, Segall illustrates how, after becoming disenchanted with the role of competition in their public health services, many OECD countries have made an explicit shift back from competition to cooperation. This is not surprising, because if one subscribes to the view that access to health care should be a human right, then it becomes patently obvious that health care would be organized in a socialized manner that equitably serves the interests of all, and “should not be left to the vagaries of the market.” ([44], p.76). Arguing against a competition-based private health system, Hunter reminds us that “[a]bandoning the public service ethos, or mission, to the vagaries of the market in the form of outsourcing public services to for-profit providers is to forget why public services came into being in the first place” ([45], p.56).

In the U.S., fierce healthcare competition has become “zero sum”, resulting in a form of “ethical fading”, as explained by Michael Porter and Elizabeth Teiberg [46]: “The system participants divide value instead of increasing it. In some cases, they may even erode value by creating unnecessary costs.” It takes the form of cost shifting rather than cost reduction, pursues greater bargaining power rather than better patient care, restricts patient choice and access to care rather than making care better and more efficient, and relies on costly litigations to settle disputes. It is no wonder that the U.S. health care system, based on a philosophy of competition, is the most expensive and yet less equitable than the Canadian system which is based more on one of cooperation. More specifically, healthcare cost per capita was $9,024 for the U.S., and $4,496 for Canada based on OECD Health Statistics 2014. While virtually all Canadians are insured for physician and hospital care, the uninsured rate among all U.S. adults was 15% in 2008, 17% in 2013, and down to 11% in the second quarter of 2016 due to Obamacare [47]. A 2009 study further estimated that this lack of health insurance was associated with approximately 45,000 deaths among adult Americans in 2005 [48]. As the University of Toronto Professor Raiser Deber stated:

Canadian health policy analysts have vehemently defended the principle of “single-tier” publicly funded medicine for “medically necessary” services, not only on the usual grounds of equity but on the grounds of economic efficiency. Multiple payers are seen not only as diminishing equity but also as increasing the burden on business and the economy to pay those extra costs. ([49], p. 20–21)

Similarly, in an ABC Radio interview in 2006, Harvard economics professor William Hsiao announced that: “The world realises they may have been following the wrong path” and “ healthcare can’t be left to the market alone… when it comes to health, the market actually leads to inflated prices.” [50] He further elaborated his view in a working paper written for the International Monetary Fund in 2007, in an effort to set the record straight about healthcare economics, and to debunk the myths related to the misconceived superiority of private-sector over public-sector health care—in terms of insurance coverage, service efficiency and quality, healthcare financing and cost. [51]

Instead of “following the wrong path” of defending competition, we suggest that the cooperative way is the right path, not only in health care but also in other spheres of human endeavours as we discuss below.

### Toward a new cooperation paradigm

*Competition has been shown to be useful up to a certain point and no further, but cooperation, which is the thing we must strive for today, begins where competition leaves off. (Franklin D. Roosevelt, Speech at the People's Forum in Troy, New York, March 3, 1912*

In his book, *Cooperation: The Basis of Sociability*, Michael Argyle defined cooperation as “acting together, in a coordinated way at work, leisure, or in social relationships, in the pursuit of shared goals, the enjoyment of the joint activity, or simply furthering the relationship.” ([52], p.4). The best research evidence to date has shown that cooperation and group effort is superior to competition and individualistic efforts, in promoting productivity and achievement on various tasks involving motor performance, verbal and spatial problem solving, concept attainment, retention and memory, and guessing, judging and predicting, etc. These results hold for all subjects (language arts, reading, math, science, social studies, psychology, and physical education) and for all age groups, as concluded by Johnson et al. [53] after conducting a meta-analysis of 122 studies. These findings have been updated and validated by Rosenau [11] and Kohn [13]. It stands to reason, therefore, that we would be better off living cooperatively.

While acknowledging that most economic models are based on the *self-interest hypothesis*, Ernst Fehr and Klaus Schmidt found “overwhelming evidence that systematically refutes the self-interest hypothesis and suggest that many people are strongly motivated by concerns for fairness and reciprocity.” They further stated ([54], p.47)

A general lesson to be drawn from these models is that the assumption that some people are fair-minded and have the desire to reciprocate does not imply that these people will always behave “fairly”. In some environments like, e.g., in competitive markets or in public good games without punishment, fair-minded actors will often behave as if they are purely self-interested. Likewise, a purely self-interested person may often behave as if he is strongly concerned about fairness like, e.g., the Proposers who make fair proposals in the ultimatum game or generous wage offers in the gift exchange game. Thus, the behavior of fair-minded and purely self-interested actors depends on the strategic environment in which they interact and on their beliefs about the fairness of their opponents.

It is critical to develop a cultural environment of cooperation in order to forestall “ethical fading” in all spheres of our lives. Furthermore, in developing or refining a paradigm of cooperation, we must differentiate key spheres of our cooperative activities upon which to focus; select and learn from best practices; and develop, amplify and multiply promising and innovative solutions.

At the individual level, a good place to start is to apply a no-contest philosophy in our daily lives, such as engaging in cooperative games and sports. In *Cooperative Games and Sports: Joyful Activities for Everyone,* Terry Orlick [55] describes over 150 field-tested activities and games for various age groups and number of players, as well as tips on how to design our own non-competitive games. As expected, research has shown that playing a cooperative game in a classroom enhances classroom interaction [56]. Similarly, students who participated in a cooperative physical education program, increased their cooperative skills and empathy, and decreased their quick-temperedness and their tendency to disrupt, compared to a control group. Moreover, students who participated in the cooperative program increased their preferences for working in groups and decreased their discomfort with group work [57].

Another way to foster cooperation is to engage in collaborative volunteerism at the local, regional, national and/or international level. The number of volunteers globally has exceeded one billion [58]. For example, in 2010, 47% of Canadians aged 15 and over contributed about 2 billion hours of their time, energy and skills to charitable and non-profit groups and organizations—a volume of work equivalent to nearly 1.1 million full-time jobs; they provided leadership on boards and committees, advocating for social or political causes, canvassing for funds, counselling or mentoring, preparing and delivering food, visiting seniors, acting as volunteer drivers, coaching children and youth, etc. Almost all (93%) cited “making a contribution to the community” as a key motivating factor in their decision to volunteer, and most also received substantial benefits, e.g., 64% said their interpersonal skills had improved [59]. As highlighted in the 2011 United Nations State of the World’s Volunteerism Report,“… volunteerism benefits both society at large and the individual volunteer by strengthening trust, solidarity and reciprocity among citizens, and by purposefully creating opportunities for participation” ([58], p.37).

At the organizational level, the best business model is unquestionably a cooperative model. The English philosopher John Stuart Mill (1806–1873), an ardent supporter of the cooperative movement, gave a most comprehensive account of why we should support cooperative institutional arrangements in Book IV, Chapter VII of his masterpiece, *Principles of Political Economy* [60]. He was in agreement with the argument put forth by Feugueray [61] that “the deepest root of evils and iniquities which fill the industrial world, is not competition, but the subjection of labour to capital, and the enormous share which the possessors of the instruments of industry are able to take from the produce.” ([60], para IV.7.64). Thus, Mill wrote of the need for a “moral revolution in society” and the benefits of cooperation in this regard ([60], para IV.7.59):

…the healing of the standing feud between capital and labour; the transformation of human life, from a conflict of classes struggling for opposite interests, to a friendly rivalry in the pursuit of a good common to all; the elevation of the dignity of labour; a new sense of security and independence in the labouring class; and the conversion of each human being's daily occupation into a school of the social sympathies and the practical intelligence.

Mill’s solutions related to two forms of partnership: (a) association of the labourers with the capitalist, and (b) association of labourers among themselves.

Among the examples he cited for his first solution was the case of a house painter in Paris, M. Leclaire, who employed about 200 workmen and paid them 4 francs for each of the 300 days of their yearly work. He assigned to himself, beside interest for his capital, a fixed allowance as manager. At the end of the year, he divided the surplus profits among all workers and himself in the proportion of their salaries. This profit-sharing scheme worked remarkably well. All the workmen earned the basic income of 1200 francs plus a minimum of 300 francs in a share of the year-end-profits. Furthermore, there were improvements in the habits and demeanour of his workmen—“not merely when at work, and in their relations with their employer, but at other times and in other relations, showing increased respect both for others and for themselves.” ^para IV.7.18^ Mill reported that other employers of labour in Paris followed Leclaire’s example on a large scale.

On the second solution, Mill has this to say:

The form of association, however, which if mankind continue to improve, must be expected in the end to predominate, is not that which can exist between a capitalist as chief, and work-people without a voice in the management, but the association of the labourers themselves on terms of equality, collectively owning the capital with which they carry on their operations, and working under managers elected and removable by themselves. ^para IV.7.21^

Mill noted that there were upwards of a hundred successful, and many eminently prosperous, associations of operatives in Paris alone. Although there was no money at all in hand and no wages could be paid at the start, these associations did not exist for the mere private benefit of the individual members, but for the promotion of the cooperative cause. Even then, Mill noted that they were already formidable competitors of the old houses, and even received complaints from a part of bourgeoisie. He was so optimistic about the future of the cooperative movement that he wrote: ^para IV.7.62^

Eventually, and in perhaps a less remote future than may be supposed, we may, through the co-operative principle, see our way to change in society, which would combine the freedom and independence of the individual, with the moral, intellectual, and economical advantages of aggregate production; and which, without violence or spoliation, or even any sudden disturbance of existing habits and expectations, would realize, at least in the industrial department, the best aspirations of the democratic spirit…

It should be noted, though, that Mill disagreed vehemently with the Socialists who argued against competition. He wrote: ^para IV.7.63^

…one of their greatest errors…is to charge upon competition all the economical evils which at present exist. They forget that wherever competition is not, monopoly is; and that monopoly, in all its forms, is the taxation of the industrious for the support of indolence, if not of plunder…

He went as far as stating that ^para IV.7.63^

even in the present state of society and industry, every restriction of it is an evil, and every extension of it, even if for the time injuriously affecting some class of labourers, is always an ultimate good. To be protected against competition is to be protected in idleness, in mental dullness…

As we argued earlier, Mill was mistaken to put his faith in competition, and minimized its negative influences in our culture and economics. However, his warning about “monopoly” and “idleness and mental dullness” must be taken seriously; it is imperative to incorporate openness and motivation for excellence in any cooperative approach to human endeavours and relations.

Next, we draw our inspirations from Karl Polyani’s *The Great Transformation*, first published in 1944 [62]. Polyani examined the social and political changes that took place in England during the rise of the market economy, and concluded that the nation state and the newly formed market economy are not separate entities but are one of human invention, “the market society”. “Economic liberalism”, Polanyi wrote, “misread the history of the Industrial Revolution because it insisted on judging social events from the economic viewpoint.” ([62], p. 35–36). He argued that if we base an economy on self-interest, then a fully self-regulating market economy will turn human beings and the natural environment into pure commodities, thus ensuring the destruction of both society and the natural environment. For Polanyi, land is simply another name for subdivided nature, labour is the day-to-day activity of human beings, and money is a token of purchasing power created and shaped by banks and governmental policies. “None of them is produced for sale” and therefore their description as commodities is purely “fictitious”^p.76^. Treating these entities as if they are “real” commodities to be bought and sold on the market, modern economic theorizing is based on a lie, and places human society at risk.

Polanyi’s argument has significant implications. The moral implication is that nature and human beings are sacred, and it is simply wrong to treat them as objects, and determine their price entirely by the market. A second implication is the central role of the state in the economy and in the management of fictitious commodities. In his view, the market society is not a naturally occurring phenomenon but a political and social construct. Even though the economy is supposed to be self-regulating, the state must play the ongoing role in the supply and management of money and credit, manpower training and unemployment insurance, food production and land-use regulations, among others. It is impossible, therefore, to sustain market liberalism’s view that the state is “outside” of the economy. Polanyi’s insights are even more salient at the international level when 60 years later we consider this statement by Kozul-Wrights and Rayment who wrote in 2004: “It is a dangerous delusion to think of the global economy as some sort of ‘natural’ system with a logic of its own: It is, and always has been, the outcome of a complex interplay of economic and political relations” ([63], p.3–4).

For Polanyi, a key step in the search for democratic alternatives, a long process, is to overturn the mindset that social life be subordinated to the market economy. He clearly admired and favoured the ideas and practices espoused by Robert Owen (1771–1858). Owenism was a forerunner of both the cooperative and the union movement:

It represented the craving of the common people…to discover a form of existence which would make man master of the machine. Essentially, it aimed at what would appear to us as a bypassing of capitalism…In spite of the machine, he believed, man should remain his own employer; the principle of cooperation or “union” would solve the problem of the machine without sacrificing either individual freedom or social solidarity, either man’s dignity or his sympathy with his fellows [62],. p.175–176

Indeed, this was the thinking behind the International Co-operative Alliance (ICA) [64], which was founded in London, England on 19 August 1895 during the first Co-operative Congress. Delegates from co-operatives from Argentina, Australia, Belgium, Denmark, England, France, Germany, Holland, India, Italy, Serbia, Switzerland, and the USA, defined and defended the Co-operative Principles and developed international cooperation and trade. Notably, the Alliance overcame political differences, and by staying committed to peace, democracy, and remaining politically neutral, was one of the only international organisations to survive both World War I and World War II. Membership in the Co-operative sector is now estimated around 1 billion, and co-operatives employ, directly or indirectly, 250 million people around the world, making up 8.73% of the employed global population.

In terms of geographic distribution, there are more than 220 million co-operatives in Asia (especially in China and India), nearly 16 million in Europe, over 7 million in Africa, over 6 million in Americas, and 60,630 in Oceania. The world's top 300 co-operatives have an estimated global turnover of 2.2 trillion USD, as revealed by the World Co-operative Monitor 2014 Report [65], which publishes results of the monitoring of the economic and social impact of cooperatives.

Cooperatives are based on the ICA’s seven principles of cooperative identity [64], which call for the practice of democracy, equality, equity and solidarity. Cooperatives also embrace the ethical values of honesty, openness, social responsibility and caring for others. Through their commitment to servicing the poor and underserved, financial cooperatives are helping to lessen the burden of poverty by providing, e.g., microfinance and medical emergencies to them. Globally, financial cooperatives reach 78 million clients living below a poverty line of $2 per day [65]. In Senegal, the health mutual, Union des Mutuelles du Partenariat pour la Mobilisation de l’Epargne et le Crédit au Sénégal (UM-PAMECAS), provides affordable insurance for saving and health care to disadvantaged and low-income families. In Peru, the Central Association of Small Producers of Organic Bananas cooperative, operating under fair trade arrangements, enables it to promote fair trade in the commercial chain and diversify the productive system in a sustainable manner. In Ethiopia, the Oromia Coffee Farmers’ Cooperative Union (OCFCU) soon realized after its establishment that its members required training in capacity building in order to succeed as coffee producers. After the union invested in training members, farmers were able to improve their production practices to win certificates in coffee quality. The union, in turn, was able to play a leading role in international coffee export. Through networking with Fair Trade Labeling Organization International, Africa Fine Coffee Association and others, the union is now exporting organic-certified coffee, fair-trade certified coffee and a host of other unique-quality products ([66], p.16–17).

Noting that a sound policy and legislative framework is required to empower cooperatives to leverage their capacity to contribute to social justice, governments adopted the United Nations guidelines on cooperatives in 2001 [64]. In 2002, governments also adopted the International Labour Organization Recommendation No. 193 [67], which emphasizes the need to promote cooperatives so they can contribute to sustainable development and decent employment to meet the urgent need for social justice. The United Nations General Assembly declared 2012 The International Year of Cooperatives as a means to raise awareness of the cooperative model, to empower cooperatives to promote their social justice values, and to encourage governments to creative supportive policy and legislative frameworks, where needed [68].

That the cooperative model is superior to other business modes in promoting social justice and equity is beyond question. Likewise, its efficiency and resilience is superior in meeting business challenges in turbulent economic environment. This was well illustrated in the case of Italy where in 2008, cooperatives already accounted for 10% of GDP and 11% of employment. During the 2007–2011 period of financial crisis, employment in cooperatives in Italy increased by 8% compared to a decrease of 2.3% in all types of enterprises. In 2012, employment in Italian cooperatives grew by a further 2.8%, adding 36,000 new jobs compared to 2011, reaching a total of 1.34 million jobs. Primarily due to social cooperatives—those involved in community services and work integration of disabled and disadvantaged people—a significant employment boom occurred between 2007 and 2011—with an increase of 17.3%, a trend which also continued in 2012 with a further increase of 4.3% [69],. p. 32.

The social cooperative movement that started in Italy to address mental health concerns, also took hold in Canada and the US in the 1980s, in the form of multi-stakeholder cooperatives, originally started as a means to fight the impact of recession [70]. The philosophy and principles upon which cooperatives operate, obviously contributes to the success of the cooperative movement, as evidenced by the fact that they now “operate in every country in the world, and in almost every kind of industry.” ([71], p.5). The success of these strategies demonstrates that cooperation is a clear alternative to competition.

At the policy level, it is important to broaden our social policy framework to capture the interplay of state, market and family [72]. First, having good government policies matters greatly, as progressive, effective distributive policies and social welfare expenditures (on health and employment insurance, education and social services, guaranteed annual income, etc.) tend to be associated with better health such as lower infant mortality rates [73]. Health care insurance policies, in particular, have profound effects on the health and health equity of their populations, e.g., in Canada and the United States. The 2016 U.S. Democratic presidential candidate Bernie Sanders’ overwhelming support from Generation Y, Millennial women (ages 18–34 in 2015) was partly attributed to his proposed, social-democratic, Canadian-style, single-payer, universal Medicare for all, high tax rates for the wealthy, and assistance to establishing worker cooperatives as a means to increase job creation and productivity in the U.S. [74, 75] In a comprehensive review of health coverage, Frenz and Vega found that “even when there is a commitment to universal coverage, the better-off almost always benefit disproportionately.” However, they also found that “free care at the point of delivery is one of the most effective facilitators for improving equity in utilization of services”, and “[r]eliance on market competition, based on patient out-of-pocket costs, appears to incur social costs and may explain persisting differences in access and utilization by social groups.” ([76], p.26–27).

Second, the win-win policies must be ‘productivist’—to actively nurture and mobilize the productive potential of the population—rather than overly relying on government benefits [72]. As the so-called ‘precarious work’—unstable, part-time, no benefits—becomes the new normal, especially among Millennials and new immigrants, public policies should vigorously promote worker cooperatives as more community-oriented and more equitable forms of business ventures than the capitalist, private firms, to help those in need of meaningful employment—e.g., older workers who have been laid off or planning career changes, and young new university graduates in search of career options. This top-down policy approach should complement bottom-up grass root initiatives, and provide the additional impetus needed to promote the cooperative movement. Indeed, the cooperative movement embodies the best of political theories also advanced by conservatives and liberals, as the successive UK governments—including the current Conservative-Liberal Coalition government—envisioned at one time a massive transformation of British public service so that one million, one in six, public sector workers would be working in public service mutuals (or cooperatives) by 2015 [77, 78]; and its failure was attributed to the inadequate policy support at both national and local levels as compared to more successful countries such as Sweden, Spain and Italy [78].

Third, these policies should promote the ethos of excellence through continuous learning and innovation. Education remains one of the most valuable investments to secure good employment and earnings [79]. In a rapidly changing world, we need to embrace the philosophy of continuous quality improvement so we may continue to excel and thrive, and not be left behind. Although cooperatives can provide friendly and supportive, ‘internal’ work environments, high performance is indispensable for both the individuals and organizations to keep flourishing in the ‘externally’ competitive world. Work enhancement and upward mobility are more likely to be realized with continual learning and innovation, in order to avoid being trapped in low-paying, unrewarding jobs for long—to the detriment of health and health equity. Thus, public policies should aim for a full-fledged lifelong learning and life-enhancing model for all, but especially for the young, so as to heavily invest in their future. Because of the likelihood of the precarity of their work, more resources should be directed to help needy young people maintain and enhance their employment—through maternity and parental leaves, adequate child benefits, better and affordable child and aged care, paid education, etc.—which would also safeguard against child poverty and ill health.

## Conclusion

Equity in health is our cherished goal. Despite numerous attempts by the international and national bodies to set targets and implement programs to achieve that goal, the progress has been uneven and not entirely satisfactory. The reason, we suggest, is that we have been reluctant to criticize the culture of competition, which engenders social inequality and health inequity.

In this paper, we have shown that avoidable and unnecessary competition is unhealthy and immoral, and we presented evidence that it inevitably leads to social inequality and health inequity. Therefore, it is imperative that we repudiate the inevitability of human competitive impulse and the so-called ‘invisible hand” to guide our economic lives. We must transcend personal and economic myths, and regain ourselves as masters of our destiny at personal, community, organizational, and policy levels. The cooperative movement, as propounded by Mill and Polanyi among others, provides us with a vision and roadmap that embodies the best of political theories advanced by distinguished conservatives, liberals and socialists.

Clearly, if our vision includes health equity and health for all, it is logical for us to choose the cooperation over the competition paradigm. Only by creating this paradigm shift will we be edging closer to our cherished vision of health and health equity for all.

### Epilogue

In *Eichmann in Jerusalem: A Report on the Banality of Evil,* the Jewish American political philosopher Hannah Arendt [80] coined a phrase, *the banality of evil*, to characterize Adolf Eichmann, a notorious Nazi war criminal responsible for shipping millions of Jewish men, women and children to their death. Writing in *The New Yorker* to report his trial in Jerusalem, Arendt was shocked to find that Eichmann was not a monster but “terrifyingly normal”, as corroborated by half a dozen psychiatrists and a minister. Her report, published in 1963, caused a storm of controversy and false accusations, led her to a sort of excommunication by the Jewish establishment in America. By ‘the banality of evil” Arendt meant not just that evil men appeared normal, but more profoundly that it was these men’s unthinkingness, thoughtlessness, and “stupidity” that allowed evil to flourish. Unquestioned obedience to a leader or faith was no defense to their actions. Although her characterization of Eichmann has been hotly contested, her concept of the banality of evil has been widely acclaimed in view of atrocities committed in more recent years by ‘ordinary’ men and women all over the world such as in Cambodia, former Yugoslavia, Rwanda, Iraq, the war on terror, etc.

Arendt’s insight reinforces our rejection of the competition paradigm. We must ask the question: How can we explain and minimize the ongoing phenomena of ‘ethical fading’? It has been manifested in everyday life, everywhere, for all genders and races, and at all ages—as seen in sibling rivalries, quarrels among friends and lovers, date rapes, internet and telephone scams and bullying, sports brawls among players and fans, hostile business takeovers, labour disputes and strikes, bank embezzlement, mass murder and terrorism, trade wars, military buildups, etc. The list is endless, and it is mostly the ordinary, normal people who have been committing these acts, often without evil intentions. We tend to act without thinking and reflecting, instead behave impulsively and get carried away by emotions. We are unwilling or unable to think critically beyond traditions and faiths—in order to engage in rational dialogues with “outsiders”. It is often due to pressures of competition that we fail to think rationally, and to resolve conflicts cooperatively. Ethics, after all, is about rational decision-making, to think and find worthy, common causes/objectives plus the best course(s) of action to achieve such objectives. Contrary to David Hume’s contention that “[r]eason is, and ought only to be the slave of the passions…” [81] we must rise above passions (or desires) to set our cherished goals such as health equity with reason if we are to behave ethically.

There is no contest between cooperation and competition in achieving health equity. As the English moral philosopher Mary Midgley states, “co-operative rather than competitive thinking always needs to be widely taught. Feuds need to be put in the background, because all students equally have to learn a way of working that will be helpful to everybody rather than just promoting their own glory. Without this, they can’t really do effective philosophy at all.” ([82], p.34). In line with Gar Alperovitz’s concept of “evolutionary reconstruction” [83], there is no quick fix, and it would take time…decades and even centuries before cooperation emerges as the dominant culture for us, if it ever will. If, and when, it happens, then we can dream about equity in health and health for all. To get there, though, it is imperative that we adopt a radically new cooperation paradigm, and apply it whenever we can and to everything we do in our daily lives.

## Abbreviations

CICOPA: International Organisation of Industrial and Service Cooperatives
CIHI: Canadian Institute for Health Information
GDP: Gross domestic product
ICA: International Co-operative Alliance
ISEqH: International Society for Equity in Health
OCFCU: Oromia Coffee Farmers Cooperative Union
UK: United Kingdom
UM-PAMECAS: Union des Mutuelles du Partenariat pour la Mobilisation de l’Epargne et le Crédit au Sénégal
US: United States
USD: United States dollar
WHO: World Health Organization
